# Noninvasive stimulation of prefrontal cortex strengthens existing episodic memories and reduces forgetting in the elderly

**DOI:** 10.3389/fnagi.2014.00289

**Published:** 2014-10-20

**Authors:** Marco Sandrini, Michela Brambilla, Rosa Manenti, Sandra Rosini, Leonardo G. Cohen, Maria Cotelli

**Affiliations:** ^1^Human Cortical Physiology and Neurorehabilitation Section, National Institute of Neurological Disorders and Stroke, National Institutes of HealthBethesda, MD, USA; ^2^Center for Neuroscience and Regenerative Medicine, Uniformed Services University of Health SciencesBethesda, MD, USA; ^3^Neuropsychology Unit, IRCCS Istituto Centro San Giovanni di Dio FatebenefratelliBrescia, Italy

**Keywords:** tDCS, aging, reconsolidation, episodic memory, prefrontal cortex, memory, enhancement, forgetting

## Abstract

Memory consolidation is a dynamic process. Reactivation of consolidated memories by a reminder triggers reconsolidation, a time-limited period during which existing memories can be modified (i.e., weakened or strengthened). Episodic memory refers to our ability to recall specific past events about what happened, including where and when. Difficulties in this form of long-term memory commonly occur in healthy aging. Because episodic memory is critical for daily life functioning, the development of effective interventions to reduce memory loss in elderly individuals is of great importance. Previous studies in young adults showed that the dorsolateral prefrontal cortex (DLPFC) plays a causal role in strengthening of verbal episodic memories through reconsolidation. The aim of the present study was to explore the extent to which facilitatory transcranial direct current stimulation (anodal tDCS) over the left DLPFC would strengthen existing episodic memories through reconsolidation in elderly individuals. On Day 1, older adults learned a list of 20 words. On Day 2 (24 h later), they received a reminder or not, and after 10 min tDCS was applied over the left DLPFC. Memory recall was tested on Day 3 (48 h later) and Day 30 (1 month later). Surprisingly, anodal tDCS over the left DLPFC (i.e., with or without the reminder) strengthened existing verbal episodic memories and reduced forgetting compared to sham stimulation. These results provide a framework for testing the hypothesis that facilitatory tDCS of left DLPFC might strengthen existing episodic memories and reduce memory loss in older adults with amnestic mild cognitive impairment.

## Introduction

Episodic memory refers to the recollection of personal experiences that contain information on what has happened and also where and when these events took place (Tulving, 1983). This form of long-term memory displays the largest degree of age-related decline (Ronnlund et al., 2005; Vestergren and Nilsson, 2010). Older adults, for example, have more difficulty recalling what they had for breakfast than do younger adults. Studies in the cognitive neuroscience of aging have begun to link declining episodic memory to neurochemical, structural and functional brain changes (Grady and Craik, 2000; Cabeza and Lennartson, 2005; Daselaar and Cabeza, 2008; Nyberg et al., 2012; Sala-Llonch et al., 2014). Because this memory is critical for daily life functioning and its decline is accelerated in conditions like amnestic mild cognitive impairment (aMCI) and Alzheimer's disease (AD), the development of effective interventions to reduce memory loss in elderly individuals and in patients with aMCI or AD is of great importance (Buschert et al., 2011; Alberini and Chen, 2012; Cotelli et al., 2012).

For more than a century, it was generally assumed that memories are unstable (i.e., susceptible to interference) for a limited-time after encoding, but as time passes, memories stabilize and become resistant to interference (McGaugh, 2000). However, this classical consolidation view has been challenged over the past 15 years by accumulating evidence showing that consolidated memories can re-enter unstable states when they are reactivated during retrieval or by a reminder cue and need to consolidate again in order to persist over longer periods of time (Nader et al., 2000a,b; Nader and Hardt, 2009; Dudai, 2012). Thus, the concept of reconsolidation assumes that memories are not consolidated once and forever, challenging the view that stability characterizes consolidated memories (Alberini and Ledoux, 2013). Indeed, memory reactivation triggers reconsolidation, a time-limited period during which the existing memory traces are vulnerable to modifications (Nadel et al., 2012; Alberini and Ledoux, 2013; Schwabe et al., 2014). There is evidence that existing episodic memories can be strengthened (Coccoz et al., 2011, 2013; Finn and Roediger, 2011; Forcato et al., 2011; Javadi and Cheng, 2013; Rodriguez et al., 2013; Sandrini et al., 2013; Bos et al., 2014), weakened/disrupted (Forcato et al., 2007; Strange et al., 2010; Chan and LaPaglia, 2013; Schwabe et al., 2013; Kroes et al., 2014), or updated by the inclusion of new information (Hupbach et al., 2007, 2008, 2009) through reconsolidation.

Clinical studies have shown that episodic memory is primarily dependent on the integrity of the medial temporal lobe. Functional neuroimaging (Fletcher and Henson, 2001; Simons and Spiers, 2003) and noninvasive brain stimulation (NIBS) studies (Manenti et al., 2012; Brem et al., 2013; Manenti et al., 2013) have also emphasized the contribution of the lateral prefrontal cortex (PFC) to episodic memory processes. In addition, evidence from neuroimaging, neurophysiology and computational modeling highlights the importance of interactions between these brain regions for memory function (Simons and Spiers, 2003).

NIBS techniques (Sandrini et al., 2011; Dayan et al., 2013), such as transcranial magnetic stimulation (TMS) and trascranial direct current stimulation (tDCS), have been used for two purposes in the study of memory function: (1) to test the causal relationship between activity in specific cortical regions and memory function; and (2) to test the general hypothesis that NIBS could modulate memory formation and learning, an issue of obvious relevance for memory research and neurorehabilitation (Zimerman and Hummel, 2010; Sandrini and Cohen, 2013, 2014).

Recent NIBS studies have showed that the dorsolateral PFC (DLPFC) plays a causal role in strengthening of verbal episodic memories through reconsolidation in healthy young adults (Javadi and Cheng, 2013; Sandrini et al., 2013).

Sandrini et al. (2013) applied repetitive TMS (rTMS) over the right DLPFC, a region critically involved in retrieval of verbal (Sandrini et al., 2003; Gagnon et al., 2010, 2011; Manenti et al., 2010) and nonverbal (Rossi et al., 2001, 2004; Gagnon et al., 2010, 2011) episodic memories. Participants learned a list of words on Day 1. On Day 2 (24h after the learning session), in a group of subjects existing memories were reactivated by a spatial-contextual reminder cue (i.e., same experimental room of Day 1) and 10 min later 1 Hz rTMS was applied to the right DLPFC. To determine whether the rTMS effect was specific to memory reactivation and relied on right PFC function, the authors designed two control groups. First, to determine whether the rTMS effect was specific to memory reactivation, they applied rTMS over the right DLPFC without memory reactivation (i.e., different experimental room), a behavioral manipulation previously successfully done in human reconsolidation studies (Hupbach et al., 2007, 2008). Second, to determine whether the rTMS effect was topographically specific, they applied rTMS over the vertex (i.e., control site) (Censor et al., 2010; Sandrini et al., 2011) after memory reactivation. Memory recall was tested on Day 3 (48 h after the learning session). The results demonstrated that rTMS over the right DLPFC after memory reactivation strengthened existing verbal episodic memories, an effect indicated by enhanced memory recall 24 h later (73%) compared to control conditions (DLPFC without memory reactivation = 56.3%; vertex-rTMS = 56.6%).

In a similar study, Javadi and Cheng (2013) applied tDCS over the left DLPFC, a region critically involved in encoding of verbal (Sandrini et al., 2003; Gagnon et al., 2010, 2011; Javadi and Walsh, 2012) and nonverbal (Rossi et al., 2001, 2004; Gagnon et al., 2010, 2011) episodic memories. Participants memorized words in the first session. Three hours later, in the second session, participants in the reconsolidation group underwent tDCS while the existing memories were reactivated during retrieval (old-new recognition task). The final session was scheduled to occur 5 h after the stimulation session. The old-new word recognition task was employed here as a measure of memory performance. The results showed that anodal tDCS enhanced episodic memory recognition compared to cathodal and sham stimulation. In order to test whether the reactivation of the existing memories was crucial for the enhancing effects of anodal tDCS, a control group did not perform the recognition task in the second session but still underwent stimulation (i.e., no reactivation condition). Contrary to the reconsolidation group, anodal stimulation did not enhance the memory performance for the control group. This result suggests that anodal tDCS over the left DLPFC enhances the reconsolidation of long-term memory only when the existing memories have been reactivated during retrieval.

Thus, the findings of these studies (Javadi and Cheng, 2013; Sandrini et al., 2013) show that noninvasive stimulation of DLPFC during reconsolidation has the potential to serve as a novel strategy to induce long-lasting memory enhancements in individuals with episodic memory decline.

In addition, tDCS over the DLPFC during retrieval enhanced episodic memory in healthy young and older adults (Manenti et al., 2013). In this study, each participant underwent two sessions of anodal tDCS (left and right) and one session of sham stimulation. The results showed that anodal tDCS applied over the left and right DLPFC induced better recognition performance in young subjects compared to sham stimulation. However, only anodal tDCS applied over the left DLPFC enhanced memory retrieval in older subjects.

Using Sandrini et al.'s paradigm (Sandrini et al., 2013) with an additional memory recall session after 30 days, the aim of the present study was to explore the extent to which anodal tDCS over the left DLPFC would strengthen existing episodic memories through reconsolidation in elderly individuals.

## Materials and methods

### Participants

Thirty-six healthy older individuals (24 females and 12 males; mean age = 67.17 ± 3.68 years; mean education = 12.05 ± 4.40 years) took part in the experiment. All of the subjects had normal or corrected-to-normal vision and were native Italian speakers (see Table 1 for demographic details). The 36 enrolled participants were randomly assigned to one of three experimental groups: Anodal-R (anodal tDCS-Reminder); Anodal-NR (anodal tDCS-No Reminder); Sham-R (sham tDCS-Reminder). tDCS was applied over the left DLPFC as in previous verbal episodic memory studies in young (Javadi and Cheng, 2013; Manenti et al., 2013) and older adults (Manenti et al., 2013).

**Table 1:** **Demographic characteristics of older individuals grouped according to the experimental conditions (Anodal-NR; Anodal-R; Sham-R), and Cognitive Reserve Index (CRI-q) and strategies questionnaire scores**.

	**Anodal-NR (*n* = 12)**	**Anodal-R (*n* = 12)**	**Sham-R (*n* = 12)**
Age (years)	67.5±2.7	67.6±4.3	66.4±4.0
Education (years)	11.8±5.0	11.3±3.9	13.2±4.4
**COGNITIVE RESERVE INDEX QUESTIONNAIRE (CRI-q)**			
CRI-total score	118.4±20.7	119.3±17.0	123.7±21.9
CRI-education	110.1±15.6	104.8±10.3	117.2±12.8
CRI-working activity	108.8±13.7	103.8±17.3	106.7±20.2
CRI-leisure time	122.7±28.9	130.0±22.1	129.7±22.1
**STRATEGIES QUESTIONNAIRE**			
Strategies total score	8.6±4.0	6.4±3.3	7.4±3.8

Participants reported being free of neurological disorders and had no history of seizures. All participants were informed about the procedures and the possible risks of tDCS, and written informed consent was obtained after a safety screening. The experimental methods got ethical approval from the local Human Ethics Committee (CEIOC) of Saint John of God Clinical Research Centre, IRCCS Istituto Centro San Giovanni di Dio Fatebenefratelli, Brescia, Italy. Prior to being enrolled in the experiment, older subjects completed a Mini Mental State Examination (MMSE) (Folstein et al., 1975) and a detailed neuropsychological evaluation to verify the absence of any cognitive deficit. A pathological score in one or more of the tests was an exclusion criterion. The neuropsychological test battery included measures used to assess non-verbal reasoning (Raven's Colored Progressive Matrices), verbal fluency (phonemic and semantic), visuo-spatial capacity (Rey-Osterrieth Complex Figure, Copy), upper-limb apraxia (De Renzi et al., 1980), attention and executive functions (Trail Making Test A and B). Moreover, memory was assessed in depth (Story Recall, Rey-Osterrieth Complex Figure Recall, Digit Span, Auditory Verbal Learning Test learning and recall). All of the tests were administered and scored according to standard procedures (Lezak et al., 2004). The results of the cognitive assessments are presented in Table 2. In addition, we administered The Cognitive Reserve Index questionnaire (CRIq), which provides a standardized measure of the cognitive reserve accumulated by individuals through their lifespan. The CRIq includes demographic data and items grouped into three sections: education, working activity and leisure time, each of which returns a subscore and compose the total score (Nucci et al., 2012) (see Table 1).

**Table 2:** **Neuropsychological assessment of older subjects grouped according to the experimental conditions (Anodal-NR; Anodal-R; Sham-R)**.

	**Anodal-NR (*n* = 12)**	**Anodal-R (*n* = 12)**	**Sham-R (*n* = 12)**	Cut off^*^
**SCREENING FOR DEMENTIA**				
Mini mental state examination	28.8±1.1	29.0±1.2	28.9±0.9	>24
**NON-VERBAL REASONING**				
Raven-colored progressive matrices	30.6±3.6	28.9±4.5	30.2±3.8	>17.5
**LANGUAGE**				
Fluency-phonemic	39.2±10.2	43.1±11.5	38.0±10.5	>16
Fluency-semantic	45.9±8.4	48.3±7.7	44.5±8.3	>24
**MEMORY**				
Digit span	5.8±0.9	5.8±1.0	6.3±0.5	>3.75
Story recall	13.6±4.2	13.3±3.9	13.9±3.0	>7.5
Rey Auditory Verbal Learning Test (Immediate recall)	45.6±6.6	48.5±9.5	46.7±6.4	>28.52
Rey Auditory Verbal Learning Test (Delayed recall)	8.8±3.4	10.5±3.2	10.1±1.3	>4.68
Rey-Osterrieth complex figure-recall	15.3±6.5	13.9±4.7	14.5±5.8	>9.46
**PRAXIS**				
Rey-Osterrieth complex figure-copy	32.4±2.7	31.6±2.8	33.6±2.1	>28.87
De Renzi ideomotor apraxia-right upper limb	70.0±1.7	69.7±1.5	69.5±2.2	>62
De Renzi ideomotor apraxia-left upper limb	70.8±1.3	70.8±1.0	70.8±1.2	>62
**ATTENTIONAL AND EXECUTIVE FUNCTIONS**				
Trail making test-A	48.8±10.1	40.3±20.0	39.8±12.8	<93
Trail making test-B	100.7±35.7	105.8±34.0	118.3±43.8	<282

* *Cut-off scores according to Italian normative data. Raw scores are reported*.

### Stimuli

We selected 20 concrete words from the “Corpus e Lessico di Frequenza dell'Italiano Scritto (CoLFIS)” (Laudanna et al., 1995). The words were balanced according to word length and to variables known to influence memory performance, i.e., word frequency, familiarity and imageability (see Appendix A for details). The selected words were highly imageable and concrete to ensure that participants knew all the words and were able to imagine them.

### Task procedure and experimental design

The experiment consisted of four sessions on four different days: Day 1 (learning session), Day 2 (24 h after learning session), Day 3 (48 h after learning session) and Day 30 (1 month after learning session). Participants were informed that they would have to memorize a list of object words and that in the second day they would receive a 15 min session of tDCS. No information was given to participants regarding the third day and the session after 1 month (i.e., recall sessions).

On Day 1, subjects (*n* = 36) were asked to learn a list of 20 object words (see Appendix A). This procedure was repeated until the participants remembered at least 17 of the 20 words (85%) or until a maximum of five learning trials was reached. The experimenter pulled out one item at a time at random (a word written on piece of cardboard) from a white bag. Participants were asked to name each word, to pay close attention so they could remember the words later and to place them in a distinctive blue bag. After all 20 words were placed into a blue bag, the experimenter took away this bag and asked the participants to remember as many words as possible. Before the next learning trial, the words were placed in the white bag again and mixed. The entire learning session took about 20–25 min to complete.

At the end of this experimental session, all 36 subjects were asked to fill in a “Memory strategies questionnaire.” This questionnaire comprised 12 possible strategies that could be used during the task and subjects had to assign a score from 1 to 10 (1 = never, 10 = always) to each strategy according to how often they had used each strategy during the task. The 12 listed strategies were: (i) to use words' initials, (ii) to create sentences including some of the presented words, (iii) to imagine the pictures corresponding to the presented words, (iv) to repeat the words, (v) to create songs including some of the presented words, (vi) to create rhymes between the displayed words, (vii) to translate the words in a foreign language, (viii) to create associations of words, (ix) to create a brief story including the presented words, (x) to associate each word to a personal event, (xi) to classify each word as easy/difficult, abstract/concrete, positive/negative, etc., (xii) to imagine the words' sound, color, shape, etc. (Manenti et al., 2010).

On Day 2 (24 h after the learning session), the procedure differed for the three experimental groups. For the Anodal-R and Sham-R groups, the experimenter, who administered the procedure in the same experimental room on Day 1, showed them the empty blue bag and asked, “Do you remember this blue bag and what we did with it yesterday?” Participants were encouraged to describe the procedure, but were stopped if they started to recall any specific words. On the basis of previous findings showing that the reconsolidation process seems to begin between 3 and 10 min after memory reactivation (Monfils et al., 2009), subjects received tDCS (anodal or sham) 10 min after the reminder. There is evidence that existing memories are automatically reactivated if the subjects are in the same experimental room of Day 1 (Hupbach et al., 2008).

For the Anodal-NR group, a new experimenter administered the experimental procedure in a different experimental room. The experimenter only applied anodal tDCS without presenting the blue bag and asking what had happened on Day 1. Day 2 session took on average 30 min to complete.

On Day 3 (48 h after the learning session), the experimenter asked the participants to recall as many words as possible from Day 1, and the experimenter noted the remembered words. When participants indicated that they could not remember any more words, the experimenter engaged the participants in a figure copying task for about 30 s. The experimenter repeated the recall test by asking the participants to recall the words again. This procedure was repeated for a total of four consecutive recall trials in order to test reliability of recall. The recall session took about 15 min to complete.

On Day 30 (1 month after learning session), the procedure was exactly the same of Day 3.

**Figure 1:**
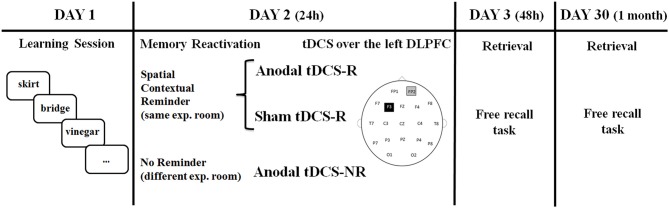
**Timeline of experiment**. Older adults learned 20 words on Day 1. On Day 2 (24 h later), they received a reminder or not, and after 10 min tDCS was applied over the left DLPFC. Memory retrieval (free recall) was tested on Day 3 (48 h later) and Day 30 (1 month later).

The experiment design is based on a previous reconsolidation study (Sandrini et al., 2013) and is illustrated in detail in Figure 1.

### tDCS procedure

tDCS is a safe, portable NIBS technique in which electrical current is directly applied to the head to generate an electrical field that modulates neuronal activity (Nitsche and Paulus, 2000; Nitsche et al., 2008; Dayan et al., 2013). Anodal tDCS has a general facilitation effect and causes membrane depolarization, whereas cathodal tDCS has a general inhibitory effect and causes membrane hyperpolarization.

A battery-driven stimulator (BrainStim, EMS, Bologna, Italy) delivered low, constant current through a pair of saline-soaked sponge electrodes (7 × 5 cm). A constant current of 1.5 mA was applied for 15 min (with a ramping period of 10 s at the beginning and end of the stimulation). The current density (0.043 mA/cm^2^) was maintained below safety limits (Poreisz et al., 2007). The electrodes were secured using elastic bands, and to reduce contact impedance, an electroconductive gel was applied under the electrodes before the montage. The study was a randomized single-blind experiment: the subjects did not know which stimulation they received, but the experimenter did. Twenty-four participants received anodal (AtDCS- NR and Anodal-R Groups) and 12 subjects received sham (StDCS–R) tDCS stimulation over the left DLPFC, a region critically involved in retrieval of episodic memories in older subjects (Manenti et al., 2013). For anodal stimulation of the left DLPFC, the anode was placed over F3 according to the 10–20 EEG international system for electrode placement, and the cathode was placed over the right supraorbital area (Herwig et al., 2003). In the sham stimulation, the tDCS montage was the same, but the current was turned off 10 s after the beginning of the stimulation (plus the duration of the fade-in = 10 s) and was turned on for the last 10 s of the stimulation period (plus the duration of the fade-out = 10 s) (see Figure 1). Therefore, subjects felt the itching sensations below the electrodes at the beginning and at the end of the stimulation, making this condition indistinguishable from the experimental stimulation (Gandiga et al., 2006). Potential tDCS side effects were assessed with a questionnaire at administered at the end of the stimulation session (Fertonani et al., 2010).

### Statistical analyses

Demographic, neuropsychological variables, tDCS sensations, cognitive reserve and strategy use were compared between the three experimental groups using parametric (*t*-test) and non-parametric (Kruskal–Wallis test) analyses were measured.

A 3×3 repeated measures ANOVA was used to analyze the mean percentage of words correctly recalled with one within-group factor of Time (Day 1, Day 3, and Day 30) and one between-group factor of Group (Anodal-NR, Anodal-R, Sham-R). *Post-hoc* analysis was carried out by Fisher's Least Significant Difference (LSD) tests for evaluating pairwise comparisons among levels of ANOVA significant factors in order to discover which of the comparisons were responsible for rejections in ANOVA test (Hayter, 1986). Statistical analyses were performed using Statistica software (version 10; www.statsoft.com).

## Results

No differences in age [*H*_(2)_ = 1.53, *p* = 0.82], education [*H*_(2)_ = 2.78, *p* = 0.60], tDCS sensations [*H*_(2)_ = 4, 9, *p* = 0.30], strategy use [*H*_(4)_ = 2.92, *p* = 0.57], and cognitive reserve [*H*_(2)_ = 0.54, *p* = 0.97] were observed between the experimental groups (see Table 1). Furthermore, no differences in neuropsychological tests were shown in the Table 2.

Perceptual sensations induced by the anodal tDCS and sham tDCS conditions were assessed with standardized questionnaire developed by Fertonani et al. (2010). Participants were asked to evaluate intensity of several perceptual sensations (i.e., itching, pain, burning, heat, pinching, iron taste, fatigue, effect on performance) through a 5-point-scale (0, none; 1, mild; 2, moderate; 3, considerable; and 4, strong). By interpreting the questionnaire completed by all subjects at the end of each type of stimulation we inferred that all the subjects tolerated well the stimulation and reported only marginal perceptual sensations. Itching and irritation were the most commonly reported perceptual sensations, with light to moderate intensity. Overall, the experienced perceptual sensations started at the beginning of the experiment and did not last long. For each group (real and sham stimulation), the sensations scores reported during anodal tDCS were compared with the sensations reported during the sham tDCS by a single-tailed independent *t*-test. These analyses showed that the anodal stimulations could not be distinguished from the sham [*H*_(2)_ = 4.9, *p* = 0.30]. Hence there are no reasons to reject the single-blinded character of this study on the basis of these results.

### Task results

In order to compare the learning rate of the three experimental groups, we recorded how many learning trials (1–6) were necessary for participants to recall at least 17 words (85%) on Day 1. Participants who recalled <17 words during the fifth learning trial were given a score of 6. Participants needed on average 4.9 (SD 1.1) learning trials to reach this criterion (Anodal-R = 5.2 SD 1.0; Anodal-NR = 4.8 SD 1.3; Sham-R = 4.8 SD 1.0). There were no significant differences between the three groups (*H* = 0.87, *p* = 0.65). Furthermore, participants recalled on average the 79.2% of the words at the last learning trial (SD 11.7) to reach this criterion (Anodal-R = 81.6% SD 12.9; Anodal-NR = 77.5% SD 11.4; Sham-R = 78.3% SD 10.9). There were no significant differences between the three groups (*H* = 1.56, *p* = 0.81).

The mean percentage of words correctly recalled was analyzed with ANOVA with “group” (Anodal-R, Anodal-NR, Sham-R) as the between subjects variable and “time” (Day 1, Day 3, and Day 30) as the within-subjects variable.

The analyses showed significant effects for “group” [*F*_(2, 33)_ = 4.64, *p* = 0.02, η^2^_*p*_ = 0.220], “time” [*F*_(2, 66)_ = 73.8, *p* < 0.001, η^2^_*p*_ = 0.691] and the interaction between “group” and “time” [*F*_(4, 66)_ = 3.56, *p* = 0.01, η^2^_*p*_ = 0.178]. Based on these results, we were interested in evaluating pairwise comparisons among levels of significant effects. In particular we wanted to highlight the performance accuracy differences from Day 1 to Day 3 and from Day 1 to Day 30 in all groups and compare performance among different groups. *Post-hoc* Analyses were performed using LSD test and all *p*-values are reported in Table 3.

**Table 3:** ***p*-Values of all *post-hoc* analyses using LSD test**.

		**Anodal-NR**			**Anodal-R**			**Sham-R**		
		**Day 1**	**Day 3**	**Day 30**	**Day 1**	**Day 3**	**Day 30**	**Day 1**	**Day 3**	**Day 30**
	Mean	77.5%	49.9%	47.2%	81.6%	49.9%	56.3%	78.3%	31.9%	24.7%
	SD	11.4	17.3	25.9	12.9	24.3	25.6	10.9	16.6	17.6
**ANODAL-NR**										
Day 1			0.000^*^	0.000^*^	0.591	0.001^*^	0.007^*^	0.914	0.000^*^	0.000^*^
Day 3		0.000^*^		0.648	0.000^*^	1.000	0.413	0.000^*^	0.022^*^	0.002^*^
Day 30		0.000^*^	0.648		0.000^*^	0.727	0.244	0.000^*^	0.051	0.005^*^
**ANODAL-R**										
Day 1		0.591	0.000^*^	0.000^*^		0.000^*^	0.000^*^	0.667	0.000^*^	
Day 3		0.001^*^	1.000	0.727	0.000^*^		0.286	0.000^*^	0.022^*^	
Day 30		0.007^*^	0.413	0.244	0.000^*^	0.286		0.005^*^	0.002^*^	0.000^*^
**SHAM-R**										
Day 1		0.914	0.000^*^	0.000^*^	0.667	0.000^*^	0.005^*^		0.000^*^	0.000^*^
Day 3		0.000^*^	0.022^*^	0.051	0.000^*^	0.022^*^	0.002^*^	0.000^*^		0.228
Day 30		0.000^*^	0.002^*^	0.005^*^	0.000^*^	0.002^*^	0.000^*^	0.000^*^	0.228	

* *p < 0.05*.

**Figure 2:**
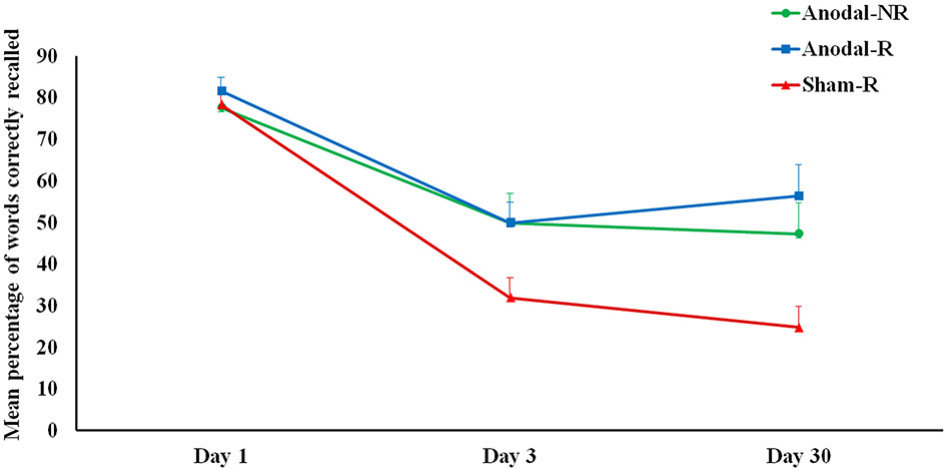
**The plot shows the mean percentage of words correctly recalled in each group (Anodal-R, Anodal-NR and Sham-R) at Day 1, Day 3, and Day 30**. There was significant memory decay (i.e., forgetting) from Day 1 to Day 3 in all groups. Forgetting was reduced up to 1 month in the anodal tDCS groups (Anodal-R and Anodal-NR) compared to the sham group (Sham-R). Error bars represent standard errors of the means (s.e.m.). ^*^p < 0.05.

Considering the group effect, the *post-hoc* comparisons showed significant differences between Anodal-NR (mean 49.9; SD 21.6) and Sham-R (mean 31.9; SD 17.1) (*p* = 0.04) and between Anodal-R (mean 49.9; SD 24.6) and Sham-R (*p* < 0.01). No difference was found between Anodal-NR and Anodal-R (*p* = 0.47) (see Figure 2).

Considering the time effect, significant differences were found between Day 1 and Day 3 (*p* < 0.01) and between Day 1 and Day 30 (*p* < 0.01). No difference was found between Day 3 and Day 30 (*p* = 0.73).

Regarding the interaction, we found significant differences in all three groups between Day 1 and Day 3 (Anodal-NR Day 1 vs. Day 3 *p* < 0.01); (Anodal-R Day 1 vs. Day 3; *p* < 0.01); (Sham-R Day 1 vs. Day 3; *p* < 0.01). Moreover, significant differences in all three groups between Day 1 and Day 30 (Anodal-NR Day 1 vs. Day 30; *p* < 0.01); (Anodal-R Day 1 vs. Day 30; *p* < 0.01); (Sham-R Day 1 vs. Day 30; *p* < 0.01).

These findings show memory decay (i.e., forgetting) from Day 1 to Day 3 and from Day 1 to Day 30 in all groups. In addition, in the Sham-R group we observed a significant decrease in memory performance at Day 3 compared to Anodal-NR (*p* = 0.02) and Anodal-R (*p* = 0.02). Moreover, we observed a significant decrease of memory performance at Day 30 in Sham-R compared to Anodal-NR (*p* < 0.01) and Anodal-R (*p* < 0.01). These results show reduced forgetting up to 1 month in the anodal tDCS groups compared to the control group (Sham-R) (see Figure 2).

## Discussion

The results of this study show that anodal tDCS over the left DLPFC (i.e., Anodal-R and Anodal-NR groups) strengthened existing memories and reduced forgetting in healthy older subjects compared to the sham group (i.e., Sham-R). Importantly, this behavioral effect was not influenced by differences between groups in the learning rate, number of words correctly recalled in the last learning trial, cognitive reserve accumulated and memory strategies used.

Previous behavioral (Hupbach et al., 2008) and rTMS (Sandrini et al., 2013) reconsolidation studies in healthy young adults have shown that under laboratory conditions the original spatial context (i.e., same experimental room of Day 1) plays a role in reactivating the existing memories. In our study involving older adults, we observed similar behavioral facilitation effects (i.e., memory strengthening) in both the anodal tDCS over the left DLPFC groups, despite participants in the Anodal-NR group were tested in a different room (i.e., different spatial context). Depending on subject population and features of the environment, memory reactivation might be triggered by other factors. Unlike the young adults (Sandrini et al., 2013), being in the same institute/center may have been more salient to older adults than the distinction between the two rooms, and the no reminder group (Anodal-NR) would have been reminded of the learning session and performed the same as the reminder group (Anodal-R). There is evidence that older adults have poor memory for the source and problems with the process of binding memories so that perceptual and contextual cues may be encoded but may not be appropriately bound to the target event (Schacter et al., 1991; Naveh-Benjamin and Craik, 1995; Chalfonte and Johnson, 1996). In addition, Kroes and colleagues using electroconvulsive therapy in patients with unipolar depression showed memory reconsolidation impairment, despite a change of room in the hospital, a finding that highlights the strength of the hospital context for allowing memory reactivation (Kroes et al., 2014). Regardless of spatial context, a previous reconsolidation study demonstrated that anodal tDCS over the left DLPFC strengthens verbal episodic memories in young subjects only when the existing memories have been reactivated (Javadi and Cheng, 2013). Although all these findings suggest that our facilitation effect observed in the both anodal tDCS groups might be triggered by the reactivation of the existing memories, further work is needed on the reconsolidation process in the elderly.

Considering the design limitations due to the fact that a sham-no reminder group was not tested, and the lack of statistical significance between Day 3 and Day 30 in all groups (see Figure 2), the behavioral effects observed at 1 month might be not due to the effect of anodal tDCS but to the repeated reactivation of the existing memories during testing (four free recall trials) on Day 3. Strengthening effects as a result of reconsolidation have been reported in animal and human studies with successive reactivations of the memories (Forcato et al., 2011; Inda et al., 2011). Inda et al. (2011), using inhibitory avoidance learning in rats, found that successive reactivations of existing memories, by re-exposition to the context, resulted in reconsolidation that mediated memory strengthening and prevents forgetting.

Previous studies have shown that anodal tDCS may enhance retrieval (Manenti et al., 2013) and consolidation (Floel et al., 2012) of episodic memories in the elderly. Manenti et al. (2013) demonstrated that anodal tDCS applied over the left DLPFC or left posterior parietal cortex during the retrieval phase enhanced recognition of verbal memories. In addition, Floel et al. (2012) showed that anodal tDCS over the right temporo-parietal cortex during an object-location task did not alter the learning rate and the immediate free recall but significantly enhanced the delayed recall (1 week) compared to sham (Floel et al., 2012). These findings further support the hypothesis (Reis et al., 2009) that there is a consolidation mechanism that is susceptible to anodal tDCS and contributes to offline effects more than to online effects.

Here, we show for the first time that anodal tDCS over the left DLPFC strengthens existing verbal episodic memories and reduces forgetting in the elderly. In addition, this study confirms the critical role of left DLPFC in verbal episodic memory along the lifespan (Gagnon et al., 2010; Manenti et al., 2010, 2011, 2013; Gagnon et al., 2011; Javadi and Walsh, 2012; Javadi and Cheng, 2013).

Regarding the putative brain mechanisms of this facilitation effect, there is evidence that tDCS affects not only the targeted local region but also activity in remote interconnected regions (Pena-Gomez et al., 2012; Venkatakrishnan and Sandrini, 2012; Saiote et al., 2013; Stagg et al., 2013). Anodal tDCS over the left DLPFC might have enhanced the functional coupling between the PFC and the hippocampus, thereby enhancing memory recollection. This speculation has also been suggested in a similar reconsolidation study using 1 Hz rTMS over the right DLPFC in young subjects (Sandrini et al., 2013). This might initially seem counterintuitive given the widely used rule of thumb that 1 Hz stimulation decreases cortical excitability, inducing inhibitory effects. However, this principle is mainly derived from basic studies of motor cortex and does not necessarily apply to other cortical regions and more complex cognitive functions (Sandrini et al., 2011). For instance, Turriziani et al. (2012) showed that 1 Hz rTMS (considered to have an inhibitory effect) of right DLPFC enhanced episodic memory while intermittent Theta Burst Stimulation (considered to have a facilitatory effect) of the same region deteriorated memory performance. In addition, there is evidence that 1 Hz rTMS may improve performance of a cognitive task by strengthening the connectivity between task-relevant brain regions depending on the functional state of the cortex at the time of stimulation (Ward et al., 2010).

Combined NIBS and neuroimaging studies (Censor et al., 2013, 2014; Macher et al., 2014; Vidal-Pineiro et al., 2014) may shed light on how functional interactions between remote but interconnected brain regions may mediate strengthening of existing memories in young and older adults.

In conclusion, anodal tDCS over the left DLPFC induces beneficial effects on verbal episodic memories in older adults, suggesting that noninvasive stimulation of this cortical region might be a novel strategy to strengthen existing memories and reduce memory loss in older adults with episodic memory impairment, such as aMCI.

### Conflict of interest statement

The authors declare that the research was conducted in the absence of any commercial or financial relationships that could be construed as a potential conflict of interest.
