# Supplementary material for: Noninvasive stimulation of prefrontal cortex strengthens existing episodic memories and reduces forgetting in the elderly
Source: Front Aging Neurosci. 2014 Oct 20;6:289. doi: 10.3389/fnagi.2014.00289 (PMC4202785; doi:10.3389/fnagi.2014.00289)
Supplement: Supplementary file 1 [file Table1.DOCX]

**Appendix A. 20 word stimuli used in experiment**

The words were extracted from the “Corpus e Lessico di Frequenza dell'Italiano Scritto (CoLFIS)” (Laudanna et al., 1995) and controlled for number of letters (minimum=5, maximum=8, mean=6.25, standard deviation SD=1.07), number of syllables (minimum=2., maximum=3, mean=2.50, SD=0.51), familiarity (mean=6.37, SD=0.26), imageability (mean=5.93, SD=0.32), concreteness (mean=6.30, SD=0.53) and word frequency (mean=51.65, SD=36.22).

| **Italian words** | **English Translation** | **Letters** | **Syllables** | **Familiarity** | **Imageability** | **Concreteness** | **Frequency** |
| --- | --- | --- | --- | --- | --- | --- | --- |
| *Aceto* | Vinegar | 5 | 3 | 6.36 | 5.61 | 6.57 | 26 |
| *Antenna* | Aerial | 7 | 3 | 6.27 | 5.55 | 6.50 | 28 |
| *Asfalto* | Asphalt | 7 | 3 | 5.95 | 5.73 | 5.73 | 53 |
| *Candela* | Candle | 7 | 3 | 6.59 | 6.30 | 6.80 | 61 |
| *Carciofo* | Artichoke | 8 | 3 | 6.36 | 6.32 | 5.05 | 22 |
| *Castagna* | Chestnut | 8 | 3 | 6.50 | 6.39 | 6.61 | 30 |
| *Freccia* | Arrow | 7 | 2 | 6.30 | 5.68 | 6.80 | 40 |
| *Fucile* | Rifle | 6 | 3 | 6.52 | 6.02 | 5.36 | 80 |
| *Gabbia* | Cage | 6 | 2 | 6.14 | 6.02 | 6.36 | 54 |
| *Gonna* | Skirt | 5 | 2 | 6.82 | 6.05 | 6.64 | 83 |
| *Laccio* | Shoelace | 6 | 2 | 6.02 | 5.20 | 5.66 | 21 |
| *Perla* | Pearl | 5 | 2 | 5.86 | 6.02 | 6.73 | 30 |
| *Piscina* | Pool | 7 | 3 | 6.64 | 6.14 | 6.59 | 72 |
| *Piuma* | Feather | 5 | 2 | 6.32 | 5.73 | 5.50 | 32 |
| *Ponte* | Bridge | 5 | 2 | 6.66 | 5.84 | 6.61 | 166 |
| *Schiuma* | Lather | 7 | 2 | 6.41 | 6.00 | 5.75 | 19 |
| *Torre* | Tower | 5 | 2 | 6.27 | 6.20 | 6.68 | 64 |
| *Valigia* | Luggage | 7 | 3 | 6.70 | 6.20 | 6.86 | 101 |
| *Vulcano* | Volcano | 7 | 3 | 6.45 | 6.09 | 6.57 | 30 |
| *Zuppa* | Soup | 5 | 2 | 6.16 | 5.45 | 6.68 | 21 |
